# P5 Mental Health Platform: A Digital Solution to Monitor Anxiety and Depression Symptoms in the General Portuguese Population

**DOI:** 10.3390/epidemiologia7020056

**Published:** 2026-04-20

**Authors:** Patrícia Soares-Coelho, Luís Jesus, Mafalda Machado-Sousa, Liliana Amorim, Sónia Ferreira, Maria Picó-Pérez, Pedro Morgado

**Affiliations:** 1 Life and Health Sciences Research Institute (ICVS), School of Medicine, University of Minho, 4710-057 Braga, Portugal; patricia.a.soares.coelho@gmail.com (P.S.-C.); a53163@alunos.uminho.pt (L.J.); id9534@alunos.uminho.pt (M.M.-S.); soniamgaf@gmail.com (S.F.); picom@uji.es (M.P.-P.); 2 PT Government Associate Laboratory ICVS/3B’s, 4710-057 Braga, Portugal; 3 2CA-Clinical Academic Center, 4710-243 Braga, Portugal; 4 Association P5 Digital Medical Center (ACMP5), 4710-057 Braga, Portugal; liliana.amorim@p5.pt; 5 Departamento de Psicología, Básica Clínica y Psicobiología, Universitat Jaume I, 12071 Castellón de la Plana, Spain

**Keywords:** digital mental health, ‘P5 mental health’ platform, anxiety symptoms, depressive symptoms, Portugal

## Abstract

**Background:** The prevalence of mental disorders, particularly anxiety and depression, has been increasing and is becoming a major public health concern in Portugal. Digital mental health solutions offer scalable and accessible tools for monitoring and managing mental health. ‘P5 Mental Health’ has been created as a platform to assess and monitor symptoms of anxiety and depression in the Portuguese population, and to offer strategies to promote well-being to support users. **Objective:** This study aims to (1) describe the P5 Mental Health platform, (2) evaluate its feasibility as a digital mental health monitoring tool, and (3) analyze trends in the prevalence and severity of anxiety and depression symptoms over a four-year period, particularly in response to major societal stressors. **Methods:** Between September 2020 and September 2024, 46,032 responses were collected from platform users. Anxiety and depression symptoms were assessed using the Generalized Anxiety Disorder-7 (GAD-7) and Patient Health Questionnaire-9 (PHQ-9) scales. Longitudinal trends were analyzed across four time periods. Welch’s ANOVA and Games–Howell post hoc tests were conducted to compare symptom severity across time, and ordinal logistic regression was used to examine the impact of time on symptom progression. **Results:** Anxiety and depression symptoms increased between 2020 and 2022, stabilized thereafter, and showed a slight decline in 2024. The proportion of users reporting moderate to severe anxiety (GAD-7 ≥ 10) rose from 30.87% in September 2020 to 66.30% in June 2022. Similarly, the prevalence of moderate to severe depressive symptoms (PHQ-9 ≥ 10) rose from 3.62% in March 2021 to 51.54% in August 2021. Despite a small decrease in 2024, symptom levels remained significantly higher than baseline levels recorded at the beginning (*p* < 0.001). A strong positive correlation was found between anxiety and depression symptoms (*r* = 0.739, *p* < 0.001), underscoring their high comorbidity. **Conclusions:** This study demonstrates the feasibility of the P5 Mental Health platform as a real-time mental health monitoring tool, particularly during periods of heightened social and economic stress. The findings highlight the need for sustained digital mental health interventions beyond crisis periods to ensure long-term engagement; however, future improvements should focus on increasing user engagement and adding personalized features to ensure long-term mental health management.

## 1. Introduction

### 1.1. Background

In Portugal, the prevalence of mental disorders has been increasing significantly, reflecting a concerning global trend. It is estimated that approximately 20% of the Portuguese population experiences some form of mental disorder each year, with anxiety and depression being the most common diagnoses [1,2]. In 2019, mental disorders accounted for 8.27% of the country’s disability-adjusted life years (DALY), a figure higher than the European average of 6.65% [1,3]. In recent years, events such as the COVID-19 pandemic and armed conflicts in Europe have further exacerbated this scenario, contributing to a rise in anxiety symptoms, now affecting between 28.8% and 45.5% of the population, and depressive symptoms, ranging from 16.1% to 23.6% [1,2,3]. The increasing prevalence of mental health disorders in Portugal highlights the need for accessible and scalable solutions, including digital platforms, to support mental well-being and improve access to mental health care [4,5].

### 1.2. The Impact of Limited Preventive Mental Health Care

Despite the high prevalence of anxiety and depressive disorders, access to timely mental health care remains limited, particularly for individuals with mild to moderate symptoms. Mental health systems frequently prioritize acute or severe cases, which delays preventive interventions and increases the risk of symptom progression [6,7]. In Portugal, long waiting times for psychiatric and psychological care within the public health system and regional inequalities in service availability further constrain access to early support [1]. These systemic barriers contribute not only to individual suffering but also to broader social and economic costs [8,9].

In this context, digital mental health solutions have emerged as a scalable complement to traditional care, offering accessible tools for symptom monitoring, self-assessment, and early support [6,8]. By reducing barriers related to stigma, geography, and waiting times, digital platforms may help address gaps in preventive mental health care, particularly during periods of heightened societal stress [9,10]. However, the availability of scientifically validated, culturally adapted digital tools for population-level mental health monitoring remains limited in Portugal, highlighting the need for context-specific solutions.

### 1.3. Digital Mental Health Platforms as a Scalable Solution

The widespread adoption of smartphones and internet-enabled devices has created new opportunities for mental health care delivery. Digital mental health platforms are emerging as effective tools for symptom monitoring, self-management, and early intervention [11,12]. In 2017, approximately 325,000 health apps were available for download, with one-third focusing on mental health [13]. However, despite their growing popularity, many lack scientific validation, raising concerns about their effectiveness, privacy, and compliance with regulatory standards [14].

While digital mental health platforms have traditionally focused on managing mental health disorders, their role in prevention and well-being promotion is gaining recognition [4]. These platforms provide dynamic features for symptom tracking, emotion regulation, and stress reduction, helping users maintain long-term mental well-being [6,8]. Their accessibility and cost-effectiveness make them particularly appealing to individuals facing barriers to traditional mental health care, such as stigma, geographic limitations, and long waiting times [5].

In Portugal, telemedicine such as Linha Saúde 24 and SNS24 provide remote access to healthcare, yet validated platforms specifically designed for continuous mental health monitoring remain limited. Existing digital tools primarily focus on managing mental health problems rather than empowering individuals to proactively maintain their well-being [1]. Addressing this gap requires the development of scientifically validated applications that integrate evidence-based mental health solutions with features that promote self-awareness, emotional regulation, and preventive care [6,8].

### 1.4. The P5 Mental Health Platform

To meet these needs, the P5 Mental Health platform was developed as a community-based digital solution to assess and monitor symptoms of anxiety and depression in the Portuguese population, while providing well-being strategies. Rather than focusing on diagnosed conditions, the platform adopts a broader, community-level approach to promote self-management and prevention. Enabling real-time symptom tracking allows individuals to monitor their emotional well-being and access personalized strategies. This study introduces the P5 Mental Health platform and evaluates its acceptability and feasibility to track trends in anxiety and depressive symptoms over time in the general Portuguese population.

## 2. Methodology

### ‘P5 Mental Health’ Digital Platform Description

‘P5 Mental Health’ is a free digital platform that was developed during the COVID-19 pandemic as part of the project “Promoting Mental Health During Pandemic” created by the Life and Health Sciences Research Institute (ICVS) of the Medical School of the University of Minho in collaboration with the P5 Digital Medicine Center. The purpose of the platform was to enable its users to assess and monitor symptoms of anxiety and depression, including tools to deal with these symptoms, mental health and wellness general strategies. The platform is available through its website (https://saudemental.p5.pt/) and its app for Android and iOS devices.

‘P5 Mental Health’ ensures strict data security and privacy compliance by storing only anonymous, non-identifiable, and encrypted event sequences on a secure cloud server. Each in-app event is assigned a randomly generated unique code along with a timestamp, ensuring that no personal or identifiable information is collected. Only individuals who were 18 years old or older had access to the platform. Ethical approval was obtained from the Ethical Committee for Life Sciences of the University of Minho (Braga, Portugal) for the present study. Electronic informed consent was obtained from all the participants before data collection. The study followed the Helsinki Declaration. To promote ease of use and safeguard user privacy, the platform did not collect any sociodemographic information, thereby avoiding the storage of personal data or the association of responses with individual user profiles.

**Figure 1:**
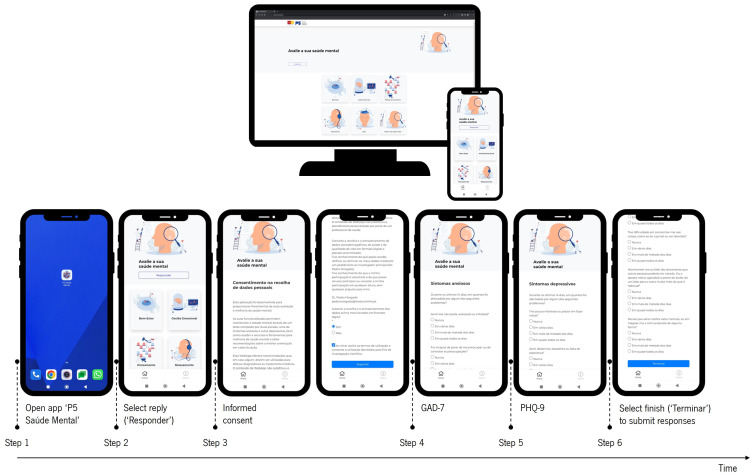
Flowchart of the response submission process on the ‘P5 Mental Health’ platform.: The workflow of the ‘P5 Mental Health’ platform is outlined as follows: Users start by opening the ‘P5 Saúde Mental’ (P5 Mental Health) app on their device (Step 1). They then select the “Responder” (answer) option to start the mental health assessment (Step 2). Before proceeding, users must read and give their consent to the collection of anonymous data (Step 3). The assessment involves completing two questionnaires: the GAD-7, which assesses anxiety symptoms (Step 4), and the PHQ-9, which assesses depressive symptoms (Step 5). After answering the questions, users select “Terminar” (finish) to submit their answers and gain access to the platform’s mental health resources (Step 6).

After accessing the ‘P5 Mental Health’ platform, each user had two options: assess his/her mental health through a brief questionnaire or directly access mental health and wellness strategies (see Figure 1). Users who chose to assess their mental health answered two self-report measures to assess anxious and depressive symptomatology. After completing and submitting the questionnaire, participants were offered mental health and wellness strategies.

Although users may complete repeated assessments, data are anonymous and not linkable to individuals; thus, analyses reflect longitudinal trends in responses rather than within-person trajectories. Since submissions cannot be linked to unique users. Analyses were therefore conducted at the response level, and repeated participation cannot be ruled out.

## 3. Measures

The Generalized Anxiety Disorder-7 [15] scale assesses anxiety symptoms through seven questions regarding experiences in the past 14 days. Each question has four response options that quantify the frequency of experienced symptoms that can range from 0 (‘Never’) to 3 (‘Almost every day’). The final score is the sum of all responses and can range from 0 to 21. Anxiety symptom severity is categorized as minimal (total score = 0–4), mild (total score = 5–9), moderate (total score = 10–14), and severe (total score = 15 or higher). Thus, only a score of 10 or higher suggests that the participant is very likely to have anxiety symptoms [15].

The Patient Health Questionnaire-9 [16] assesses depressive symptoms through nine questions regarding experiences over the past 14 days. Each question has four response options that measure the frequency of symptoms experienced on a scale between 0 (‘Never’) and 3 (‘Almost every day’). The final score is obtained by summing all answers and can range from 0 to 27. Depression symptom severity is categorized as minimal (total score = 0–4), mild (total score = 5–9), moderate (total score = 10–14), moderately severe (total score = 15–19), and severe (total score = 20 or higher). So, only a score of 10 or higher suggests that the participant is very likely to have symptoms of depression [16].

### 3.1. Mental Health and Wellness Strategies

Mental health and wellness strategies are available after the anxiety and depression assessment section of P5 Mental Health or in a section of the platform that users can access directly (i.e., without completing the assessment). All strategies can be accessed at any time by platform users.

If users experience severe symptoms of anxiety (GAD-7 ≥ 15) or at least moderately severe symptoms of depression (PHQ-9 ≥ 15), they will receive a warning suggesting that it may be important to seek professional help. The alert provides recommendations for available support options, including free services within the Association P5 Digital Medical Center (ACMP5) and mental health services offered by the Portuguese National Health System.

The mental health and wellness strategies available on the platform are described in the supplementary material (see Table S1).

### 3.2. Statistical Analysis

Statistical analyses were performed with Statistical Package for Social Science software (IBM SPSS^®^) version 28 and the JASP software (version 0.16.4.0; JASP Team, University of Amsterdam, Amsterdam, The Netherlands). *p*-values below 0.05 were considered statistically significant.

Absolute and relative frequencies were calculated to examine patterns of engagement with the P5 Mental Health platform. The normality of the quantitative variables was assessed prior to conducting inferential statistical analyses. Normality was assessed using measures of skewness and kurtosis, the Kolmogorov–Smirnov test, and visual inspection of histograms and Q-Q plots.

Descriptive statistics were presented as means, standard deviations, and confidence intervals (95% CI) for continuous variables, and absolute and relative frequencies for categorical variables. Total scores were computed only for complete item responses; incomplete submissions were excluded from analyses requiring total scores. Valid sample sizes are reported separately for GAD-7 and PHQ-9.

Welch’s ANOVA was used to assess changes in anxiety and depressive symptoms over time due to the violation of homogeneity of variances as indicated by Levene’s test. Effect sizes were reported using η^2^ (eta squared) to quantify the proportion of variance explained by time. Where significant differences were found, Games–Howell post hoc tests were used to identify pairwise differences between time periods. No additional correction for multiple comparisons was applied beyond Games–Howell post hoc testing; pairwise *p*-values are interpreted alongside effect sizes and confidence intervals.

In addition, ordinal logistic regression was used to examine the effect of time on the severity of anxiety and depressive symptoms, with pseudo-R^2^ values (Cox & Snell, Nagelkerke and McFadden) reported to assess model fit. Regression coefficients (β), standard errors (SE), Wald statistics, odds ratios (Exp(B)), and corresponding confidence intervals were reported.

Finally, Pearson correlation analysis was performed to assess the association between anxiety (GAD-7) and depressive symptoms (PHQ-9) over different time periods. Correlations were interpreted according to standard guidelines, and separate analyses were performed for each year to determine variations in the strength of the relationship over time.

Because submissions are anonymous and cannot be linked to unique individuals, mixed-effects (within-person) modeling was not feasible. Future platform iterations enabling privacy-preserving linkage would support mixed-effects and time-series approaches.

## 4. Results

### 4.1. Patterns of Use of the ‘P5 Mental Health’ Platform

**Figure 2:**
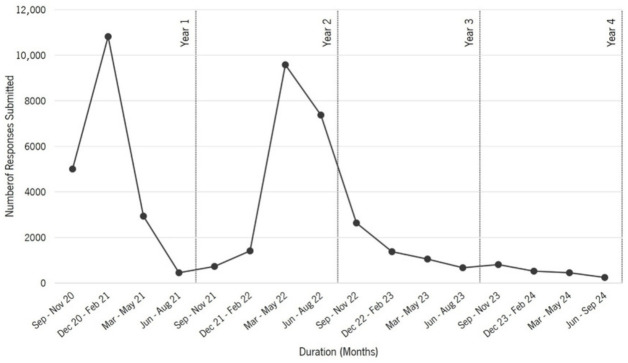
Longitudinal Trends in User Engagement on the ‘P5 Mental Health’ Platform: Number of Responses Submitted Over Time (1 September 2020–30 September 2024; *n* = 46,032). Legend: This figure shows the number of responses submitted to the ‘P5 Mental Health’ platform over time, from September 2020 to September 2024. The x-axis represents the duration in months, while the y-axis shows the number of responses submitted.

From 1 September 2020 to 30 September 2024, 46,032 responses were submitted to the P5 Mental Health platform (see Figure 2 and Table S2 for detailed information).

At the launch of the platform in September 2020, engagement peaked at 4100 responses (8.9%), but quickly declined in the following months, with 708 (1.5%) in October and 195 (0.4%) in November.

Between December 2020 and February 2021, there was a large spike, with a total of 10,820 responses (23.5%), largely driven by February (*n* = 10,556; 22.9%), the highest monthly engagement recorded.

Between March and May 2021, engagement dropped significantly to 2934 responses (6.4%), most of them in March (*n* = 1964; 4.3%). The decline continued in June–August 2021, with only 490 responses (1.1%), with the lowest number recorded in July (*n* = 126; 0.3%).

From September to November 2021, the number of responses remained low at 571 (1.2%), with a slight increase in October (*n* = 272; 0.6%). A slight recovery followed in December 2021–February 2022 with 1415 responses (3.1%), but without significant peaks.

A strong increase was observed in March–May 2022 with a total of 9008 responses (19.6%), driven by March (*n* = 7179; 15.6%). The high engagement continued in June–August 2022 with 6336 responses (13.8%), mainly from June (*n* = 5360; 11.6%).

A steady decline followed from September 2022 to April 2023, with the last significant peak in October 2022 (*n* = 1297; 2.8%). From January to April 2023, responses fluctuated between 370 and 610 per month (≤1.3%).

From May 2023 onwards, engagement remained low, with the lowest numbers recorded in July 2023 (*n* = 198; 0.4%) and July 2024 (*n* = 49; 0.1%). There was a slight increase in October 2023 (*n* = 429; 0.9%), but by mid-2024, engagement fell below 150 responses per month (≤0.3%), reaching its lowest level in August-September 2024 (≤0.1%).

The most significant peaks of responses were concentrated in February 2021 (*n* = 10,556; 22.9%), March 2022 (*n* = 7179; 15.6%), and June 2022 (*n* = 5360; 11.6%). Conversely, the lowest level of engagement was recorded in July 2024 (*n* = 49; 0.1%).

### 4.2. Monitoring Anxiety Symptoms: Longitudinal Patterns from the ‘P5 Mental Health’ Platform

**Figure 3:**
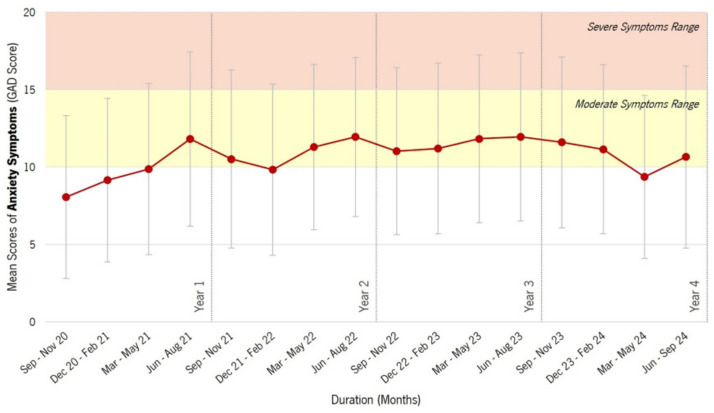
Longitudinal Trends in Anxiety Symptoms (GAD-7) on the ‘P5 Mental Health’ Platform. Legend: This figure shows the mean scores of anxiety symptoms (measured by the GAD-7 scale) among users of the P5 Mental Health platform over time. The x-axis represents the duration in months, while the y-axis shows the mean GAD-7 scores. The red line with data points shows the mean GAD-7 scores over different time periods, with error bars representing standard deviations.

When analyzing anxiety symptoms over time, the mean GAD-7 scores showed a general increase from September 2020 to mid-2022, followed by relative stability and a slight decline in 2024 (see Figure 3).

To ensure clarity and smooth progression of ideas, the period from 1 September 2020 to 31 August 2021 will be referred to as ‘Year 1’. The subsequent period from 1 September 2021 to 31 August 2022 will be referred to as ‘Year 2’. Similarly, the period from 1 September 2022 to 31 August 2023 will be referred to as ‘Year 3’, while the final period from 1 September 2023 to 30 September 2024 will be referred to as ‘Year 4’. These classifications are used consistently throughout the text to facilitate comparisons between different time periods.

The background shading highlights different ranges of symptom severity: (i) Yellow area: Moderate anxiety symptoms (GAD-7 score between 10 and 14); and (ii) Orange area: Severe anxiety symptoms (GAD-7 score of 15 or more).

Descriptive statistics (see Table 1) show that Year 1 had the lowest mean GAD-7 score (M = 9.02, SD = 5.36) of all time periods. In Year 2, there was a significant increase in anxiety symptoms (M = 11.41, SD = 5.32), which was the highest level observed over the entire study period. This trend remained relatively stable in Year 3 (M = 11.32, SD = 5.44), with only a slight decrease. In Year 4, mean anxiety scores showed a slight decrease (M = 10.88, SD = 5.55), suggesting a possible reduction in anxiety levels over time.

The graphical representation in Figure 3 is consistent with these findings, showing a steady increase in GAD-7 scores from 2020 to mid 2022, with peaks in Years 2 and 3. The slight downward trend in year 4 may indicate early signs of improvement in anxiety symptoms. However, scores remain elevated compared to Year 1, suggesting that overall anxiety levels have not returned to their initial lower levels.

### 4.3. Welch’s ANOVA Analysis

A Welch’s ANOVA was performed to compare GAD-7 scores across four time periods from September 2020 to September 2024, accounting for heterogeneity in variances. The results indicated a significant effect of time on anxiety scores, Welch’s F (3, 9650) = 552.64, *p* < 0.001, η^2^ = 0.044, suggesting that anxiety levels varied significantly across years. The effect size (η^2^ = 0.044) corresponds to a small-to-moderate effect, indicating that time explained a modest but meaningful proportion of variance in anxiety symptoms (Table 2).

### 4.4. Post Hoc Comparisons

Given the violation of homogeneity of variances (Levene’s test: F (3, 43559) = 9.85, *p* < 0.001), Games–Howell post hoc comparisons were used to examine pairwise differences.

The results showed that year 1 had significantly lower GAD-7 scores compared to years 2, 3, and 4 (*p* < 0.001), suggesting that anxiety symptoms were lower during the first year of the study. However, there was no significant difference between year 2 and year 3 (*p* = 0.693), suggesting that anxiety levels remained relatively stable between these periods. Year 3 had slightly higher anxiety levels than year 4 (*p* = 0.013), although the effect size was small, suggesting a possible decrease in anxiety symptoms in the final period (Table 3).

Overall, the results suggest that GAD-7 scores increased significantly between Year 1 and later years, indicating a possible increase in anxiety levels over time.

## 5. Effect of Time on Anxiety Symptoms: Ordinal Logistic Regression Analysis

An Ordinal Logistic Regression was used to examine the relationship between time (four time periods; September 2020–September 2024) and anxiety severity (measured by the GAD-7 scale).

The model fit was statistically significant, *χ*^2^ (3) = 2076.35, *p* < 0.001, indicating that the model effectively distinguished between levels of anxiety symptoms based on time. The pseudo R-squared values (Cox and Snell = 0.047, Nagelkerke = 0.047, McFadden = 0.008) suggest that time alone explains a small proportion of the variance in anxiety severity.

In terms of individual predictors, the transition from Year 1 to Year 2 showed a significant increase in anxiety severity (b = 0.167, SE = 0.041, Wald = 16.47, *p* < 0.001), with a positive relationship indicating that anxiety symptoms were more likely to be severe in Year 2 than in Year 1. Similarly, the transition from Year 1 to Year 3 also showed a significant increase in anxiety severity (b = 0.140, SE = 0.045, Wald = 9.48, *p* = 0.002), although the effect was slightly smaller.

However, the comparison between Year 3 and Year 4 showed a small but significant decrease in anxiety symptoms (b = −0.140, SE = 0.045, Wald = 9.48, *p* = 0.002), suggesting a possible reduction in anxiety severity in the most recent period.

In conclusion, the results of the ordinal logistic regression analysis indicate that anxiety symptoms increased significantly between year 1 and later years, with the largest increase occurring between 2020 and 2022. However, there was a slight but significant decrease in anxiety severity in the final period (2023–2024), suggesting a possible improvement in mental health outcomes over time.

### 5.1. Monitoring Depressive Symptoms: Longitudinal Patterns from the ‘P5 Mental Health’ Platform

**Figure 4:**
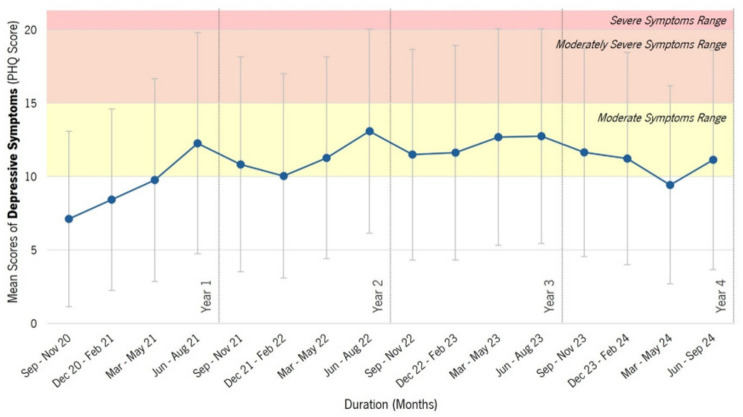
Longitudinal Trends in Depressive Symptoms (PHQ-9) on the ‘P5 Mental Health’ Platform. Legend. This figure shows the mean scores of depressive symptoms (measured by the PHQ scale) among users of the P5 Mental Health platform over time. The x-axis represents the duration in months, while the y-axis shows the mean GAD-7 scores. The blue line with data points shows the mean PHQ-9 scores over different time periods, with error bars representing standard deviations. The background shading highlights different ranges of symptom severity: (i) Yellow area: Moderate anxiety symptoms (PHQ-9) score between 10 and 14); (ii) Orange area: Moderately severe depressive symptoms (PHQ-9 score between 15 and 19); and (iii) Red area: Severe depressive symptoms (PHQ-9 score of 20 or more).

When examining depressive symptoms over time, PHQ-9 scores showed an increasing trend from September 2020 to mid-2022, followed by a period of relative stability and a slight decline in 2024 (see Figure 4).

The patterns observed in depressive symptoms closely mirror those found in anxiety symptoms. As shown in Table 4, Year 1 had the lowest mean PHQ-9 score (M = 8.37, SD = 6.36), followed by a marked increase in Year 2 (M = 11.87, SD = 7.00), which remained elevated in Year 3 (M = 11.90, SD = 7.28). A slight decrease was observed in Year 4 (M = 10.98, SD = 5.55), suggesting a modest reduction in depressive symptoms over time.

Figure 4 aligns with these trends, illustrating a rise in PHQ-9 scores from 2020 to mid-2022, stabilization in Years 2 and 3, and a small decline in Year 4. However, depressive symptom levels remain above those recorded in Year 1, indicating a lasting impact of psychological stressors throughout the study period.

### 5.2. Welch’s ANOVA Analysis

A Welch’s ANOVA was conducted to examine differences in PHQ-9 scores across four time periods from September 2020 to September 2024, adjusting for heterogeneity in variances. The analysis revealed a significant effect of time on depressive symptoms, Welch’s F (3, 7898.668) = 977.344, *p* < 0.001, η^2^ = 0.060, indicating that PHQ-9 scores varied significantly across the study period. The effect size (η^2^ = 0.060) corresponds to a moderate effect, indicating that time accounted for a modest proportion of variance in depressive symptom severity (Table 5).

### 5.3. Post Hoc Comparisons

Due to homogeneity of variances violations (Levene’s test: F(3, 44672) = 9.85, *p* < 0.001), the Games–Howell post hoc test was used to assess pairwise differences in depressive symptoms across time periods.

Results indicated that year 1 had significantly lower PHQ-9 scores compared to years 2, 3, and 4 (*p* < 0.001), reflecting an increase in depressive symptoms after the first year of the study. Notably, there was no significant difference between years 2 and 3 (*p* = 0.997), suggesting that depressive symptoms stabilized at high levels over these periods. However, a small but significant difference was observed between year 3 and year 4 (*p* < 0.001), with slightly lower PHQ-9 scores in Year 4, possibly indicating a modest improvement in depressive symptoms over time.

Overall, these results suggest that depressive symptoms peaked between years 2 and 3, followed by a slight decline in year 4. Nevertheless, the overall mean PHQ-9 scores remained higher in Year 4 than in year 1, reinforcing the idea that depressive symptoms did not return to their initial lower levels, highlighting persistent distress throughout the study period (see Table 6).

## 6. Effect of Time on Depressive Symptoms: Ordinal Logistic Regression Analysis

An ordinal logistic regression was conducted to examine the relationship between time (four time periods; September 2020–September 2024) and depressive symptom severity (measured by the PHQ-9 scale). The model fit was statistically significant, *χ*^2^ (3) = 2839.69, *p* < 0.001, indicating that time effectively differentiated levels of depressive symptoms. However, the pseudo R-squared values (Cox and Snell = 0.062, Nagelkerke = 0.062, McFadden = 0.010) suggest that time alone accounts for a modest proportion of the variance in depressive severity.

Analyzing individual time periods, the transition from Year 1 to Year 2 was associated with a significant increase in depressive symptoms (b = 0.241, SE = 0.045, Wald = 35.15, *p* < 0.001), indicating a heightened likelihood of more severe depressive states. A similar but slightly smaller increase was observed between Year 1 and Year 3 (b = 0.232, SE = 0.045, Wald = 26.59, *p* < 0.001).

In contrast, the shift from Year 3 to Year 4 demonstrated a slight but significant reduction in depressive symptoms (b = −0.232, SE = 0.045, Wald = 26.59, *p* < 0.001), suggesting a possible trend toward symptom improvement.

Overall, these findings indicate that depressive symptoms intensified significantly between 2020 and 2022, remained relatively stable in 2023, and showed a slight decline in the final period. While this downward trend may signal early signs of improvement, depressive severity remains elevated compared to Year 1, highlighting the sustained psychological impact observed across the study period.

### Association Between Anxiety and Depression

Pearson correlation analysis was used to examine the relationship between anxiety and depressive symptoms over the four-year period. The results indicated a strong positive correlation between GAD-7 and PHQ-9 scores, *r* = 0.739, *p* < 0.001, suggesting that higher levels of anxiety were significantly associated with increased depressive symptoms (see Table 7).

To assess whether this relationship varied over time, separate correlation analyses were conducted for each year. In Year 1, the correlation between GAD-7 and PHQ-9 was *r* = 0.736, *p* < 0.001, indicating a strong association between anxiety and depressive symptoms. In Year 2, the correlation decreased slightly to *r* = 0.716, *p* < 0.001, suggesting a slight reduction in the strength of the relationship between anxiety and depression. However, in Year 3 the correlation increased again to *r* = 0.728, *p* < 0.001, and remained relatively stable in Year 4 at *r* = 0.718, *p* < 0.001.

Although the strength of the correlation remained relatively stable across the years, a slight decrease in Year 2 suggests that the relationship between anxiety and depression may fluctuate slightly over time. However, the overall pattern suggests that individuals with high anxiety symptoms were likely to report high depressive symptoms consistently across all four years.

## 7. Discussion

The global increase in mental health concerns, particularly symptoms of anxiety and depression, underscores the pressing need for accessible and scalable solutions [2,17]. The ‘P5 Mental Health’ platform provides an innovative response, combining cost-effective and validated tools to support large-scale mental health monitoring [18,19]. This study analyzed engagement with the P5 Mental Health platform and longitudinal trends in anxiety and depression symptoms in Portugal between September 2020 and September 2024.

The results showed a significant increase in anxiety and depression levels between 2020 and 2022, followed by a period of stabilization and a slight decrease in 2024. However, symptoms remained elevated compared to baseline levels recorded in Year 1, suggesting that the long-term psychological impact of pandemic-related stressors and broader societal challenges persists. Interestingly, anxiety and depressive symptoms showed highly similar temporal patterns; however, in the absence of detailed sociodemographic information, it remains unclear whether this synchrony reflects a general psychological process or patterns specific to particular subgroups.

In addition, a strong positive correlation was observed between anxiety and depression symptoms (*r* = 0.739, *p* < 0.001), consistent with existing literature on the high comorbidity of these conditions [20,21]. Although strong correlations support high symptom co-occurrence, we did not model comorbidity patterns or transitions between anxiety and depression severity classes. Future work using linkable longitudinal data could examine joint trajectories and comorbidity dynamics.

These findings highlight the critical role of digital platforms in improving mental wellbeing by providing accessible and quantifiable resources for thorough mental health assessment [19,22], as well as the need for integrated mental health solutions that simultaneously address anxiety and depression symptoms.

### 7.1. Digital Mental Health and Benefits of the P5 Platform

Digital platforms offer a scalable solution to overcome traditional barriers to mental health care, such as geographic limitations, financial constraints, and stigma. The P5 Mental Health platform provides free, anonymous, and remote access to mental health resources, facilitating continuous monitoring of anxiety and depression symptoms [23,24]. The high engagement, with nearly 46,000 responses in four years, demonstrates its broad reach, supporting both individuals with significant symptoms and those seeking preventive care [25,26]. Post-crisis engagement decay is common in digital mental health platforms. This supports the need for sustained engagement strategies (e.g., personalization, reminders, linkage to care pathways) to maintain long-term monitoring.

Unlike commercial international platforms (e.g., Woebot, Headspace, BetterHelp, Talkspace), P5 Mental Health was developed as a non-commercial, population-level monitoring and self-assessment tool tailored to the Portuguese context, rather than as a subscription-based therapeutic service. Although these platforms offer mental health assistance, they lack cultural and linguistic adaptation for Portugal and do not incorporate standardized measures for the Portuguese population like the GAD-7 and PHQ-9 [27,28]. When it was launched, there were no similar digital tools available in Portugal, and the P5 Mental Health platform was quickly developed in response to the crisis brought on by the pandemic to provide a validated, evidence-based resource.

Beyond symptom tracking, P5 Mental Health provides a secure, stigma-free space for mental health self-assessment, while also allowing professionals to monitor symptom trends, supporting personalized medicine [23]. However, long-term engagement remains a challenge, as usage peaked during crises but declined afterward. Future improvements should incorporate gamification features, personalized notifications, and recommendations to sustain engagement [25,26]. While P5 Mental Health meets high data security standards (GDPR-compliant), continuous innovation in engagement strategies will be essential to maximize its long-term impact as an accessible and effective digital mental health resource in Portugal.

We highlight that this study evaluates platform feasibility and symptom monitoring trends; it does not assess the effectiveness of individual wellness strategies. Future studies should examine how engagement with specific features relates to symptom outcomes.

### 7.2. Longitudinal Trends in Anxiety and Depression Symptoms

The increase in anxiety and depression symptoms from 2020 to 2022 aligns with global studies highlighting the psychological burden associated with significant societal disruptions [29,30]. Previous research has shown that various stressors— including social isolation, financial instability, and health concerns—have contributed to a widespread decline in mental well-being [31,32]. Because P5 Mental Health was launched during the COVID-19 pandemic, no pre-pandemic baseline exists; accordingly, Year 1 should be interpreted as an internal reference period rather than a true baseline. Nevertheless, these findings underscore the need to develop proactive mental health initiatives aimed at promoting well-being and equipping individuals with tools for symptom management.

Although symptoms declined slightly by 2024, they remained above pre-pandemic levels, indicating long-term psychological distress [33,34]. This suggests that chronic stressors, such as economic uncertainty and geopolitical instability, may contribute to ongoing mental health challenges.

Symptoms of anxiety and depression increase steadily over time, with 30.87% of users reporting moderate to severe anxiety in September 2020, peaking at 22.9% of total responses in February 2021, and rising further to 66.30% in June 2022. Spikes in engagement in March 2022 (15.6%) and June 2022 (11.6%) suggest continued distress after the pandemic. Depressive symptoms followed a similar pattern, with 51.54% of users reporting depressive symptoms in August 2021, reaching 11.87 (SD = 7.00) in Year 2 and 11.90 (SD = 7.28) in Year 3. A slight decrease was observed in year 4 (M = 10.98, SD = 5.55), suggesting an early sign of improvement, but still above pre-pandemic levels. Similar trends have been reported in studies of mental health apps, highlighting a global increase in distress during crises, followed by a gradual recovery [19,22]. In the national context, digital platforms such as P5 Mental Health are essential for providing well-being strategies and mental health monitoring, which can be fundamental in reducing long-term psychological distress and enhancing mental health resilience [1].

### 7.3. Societal Influences on Symptom Trends

Mental health trends cannot be attributed solely to the pandemic. Economic uncertainty, prolonged social disruption, and geopolitical challenges contributed to persistent mental health struggles [17,23]. Studies indicate that successive lockdowns, financial instability, and social isolation had a cumulative psychological toll, exacerbating symptoms over time [35,36].

Beyond the pandemic, broader societal stressors may have contributed to sustained symptom severity. Economic instability temporally overlapped with later periods of elevated symptom burden [28,37]. While causal inference is not possible, similar associations between macro-level stressors and digital mental health engagement have been described in prior work [22,25].

### 7.4. Practical Suggestions for Future Development

For researchers and developers aiming to advance similar platforms, several strategies can enhance their feasibility. Collaborating with healthcare providers ensures that users can transition seamlessly from self-monitoring to professional care, creating a comprehensive support network. Offering tools in multiple languages broadens accessibility and usability for diverse populations, enabling a more inclusive approach. Providing users with options to personalize their experience through tailored notifications, goal tracking, and adaptive interventions can significantly improve engagement and adherence. Recent AI-enabled platforms further illustrate the potential for personalization in digital mental health [38]. Leveraging aggregated and anonymized data can support the development of public health strategies and inform resource allocation based on observed trends and needs. Strengthening data security and ensuring compliance with relevant data protection regulations, such as the General Data Protection Regulation, builds user trust and promotes long-term retention.

## 8. Limitations and Future Directions

The present study has certain limitations. Key limitations include: (i) self-selection due to open access; (ii) absence of sociodemographic variables limiting subgroup analyses; (iii) lack of a pre-pandemic baseline; (iv) no external comparison group; (v) anonymous, response-level data that cannot rule out repeated submissions; and (vi) potential confounding by unmeasured factors (e.g., media exposure, concurrent policy changes). These limitations may bias prevalence estimates and restrict causal interpretation as we expand below.

Although the statistical analyses control differences in group sample sizes, the open-access nature of the platform prevents control over sample numbers and characteristics. The open-access design may introduce selection bias, as individuals experiencing psychological distress may be more likely to use the platform. The study also lacks an external comparison group; therefore, it is not possible to determine whether observed temporal changes are specific to platform users or reflect broader population-level changes. Therefore, prevalence estimates should be interpreted as reflecting symptom burden among platform users rather than the general Portuguese population. Additionally, attrition bias is possible, as continued platform use may differ systematically by symptom severity, potentially influencing observed trends over time. Future studies could address this by implementing recruitment strategies that aim for more diverse user participation or by weighing the data to account for overrepresented groups.

Furthermore, the development of the ‘P5 Mental Health’ platform in response to the first wave of the pandemic in Portugal meant that a pre-pandemic comparison was not possible. Additionally, the lack of socio-demographic data collected, such as age, gender, and location, limits the statistical analyses conducted and the potential for generalizing the findings. This prevents stratified analyses (e.g., by age or gender) and may mask heterogeneity across population subgroups. Future platform versions should consider collecting a minimal, non-identifiable set of sociodemographic variables to enable subgroup analyses while preserving low-barrier access. Collecting such data in future iterations could also provide valuable insights into the platform’s reach and efficacy across different population segments.

Finally, as a cross-sectional study, it does not allow for the tracking of users over time, but only the analysis of associations between the variables studied. Given these limitations outlined, it would be beneficial in the future to include a short socio-demographic questionnaire in the platform and to replace the automatically generated user code with a code entered by the user according to a suggested structure (e.g., first initial of their first and last name, followed by the last four digits of their telephone number). This approach would maintain the anonymity of the data while providing insight into how often and at what times the same user has accessed the ‘P5 Mental Health’ platform.

## 9. Conclusions

This study supports the feasibility of P5 Mental Health as a digital tool for large-scale monitoring of anxiety and depressive symptoms in Portugal, showing increased symptom burden during 2020–2022 with subsequent stabilization and slight decline by 2024.

However, findings should be interpreted in light of key limitations, including the open-access, self-selected sample, lack of sociodemographic data, absence of a pre-pandemic baseline and external comparison group, and response-level anonymity. Future work should incorporate privacy-preserving linkage and minimal sociodemographic measures to enable subgroup analyses, model individual trajectories, and evaluate the impact of specific platform features on outcomes.

## Figures and Tables

**Table 1:** Descriptive Statistics for GAD-7 Scores Across Time Periods (September 2020–September 2024; *n* = 43,563).

Time Period	*n*	Percentage (%)	Mean (SD)	Range	95% CI Lower	95% CI Upper
Year 1	18,224	41.8	9.02 (±5.363)	38	8.94	9.09
Year 2	17,850	41.0	11.41 (±5.315)	30	11.33	11.49
Year 3	5515	12.7	11.32 (±5.442)	22	11.17	11.46
Year 4	1974	4.5	10.88 (±5.551)	32	10.63	11.12

*n*: Sample size; SD: Standard deviation; CI: Confidence interval (95%). Mean (SD) represents the average GAD-7 score with its standard deviation. The 95% CI is the range within which the true population mean is expected to fall with 95% confidence.

**Table 2:** ANOVA and Welch’s ANOVA Results for GAD-7 Scores Across Time Periods (September 2020–September 2024; *n* = 43,563).

Source	Sum of Squares	df	Mean Square	F	Sig.	η^2^
Between Groups	58,149.494	3	19,383.165	674.073	<0.001	0.044
Within Groups	1,252,551.794	43,559	28.755	-	-	-
Total	1,310,701.288	43,562	-	-	-	-
	Welch’s F		df _1_	df _2_		Sig.
Welch	675.745		3	7787.195		<0.001

Note. df: Degrees of Freedom; F: F-ratio; Sig.: *p*-value indicating statistical significance; η^2^: Eta squared, a measure of effect size. df _1_ represents the degrees of freedom for the numerator (number of groups minus one), while df _2_ is adjusted based on the variance and sample sizes in each group.

**Table 3:** Post Hoc Comparisons of GAD-7 Scores Across Time Periods Using Games–Howell Test.

Comparations	Mean Difference	SE	Sig.	95% CI Lower	95% CI Upper
Year 1–Year 2	−2.393	0.056	<0.001	−2.54	−2.25
Year 1–Year 3	−2.302	0.083	<0.001	−2.52	−2.09
Year 1–Year 4	−1.862	0.131	<0.001	−2.20	−1.52
Year 2–Year 3	0.091	0.083	0.693	−0.12	0.31
Year 2–Year 4	0.531	0.131	<0.001	0.19	0.87
Year 3–Year 4	0.044	0.145	0.013	0.07	0.81

Note. SE: Standard error of the mean difference; Sig.: *p*-value indicating statistical significance; CI: Confidence interval (95%). The 95% CI is the range within which the true population mean is expected to fall with 95% confidence.

**Table 4:** Descriptive Statistics for PHQ-9 Scores Across Time Periods (September 2020–September 2024; *n* = 44,676).

Time Period	*n*	Percentage (%)	Mean (SD)	Range	95% CI Lower	95% CI Upper
Year 1	18,873	42.2	8.37 (±6.360)	54	8.28	8.46
Year 2	18,135	40.6	11.87 (±7.002)	48	11.77	11.98
Year 3	5648	12.6	11.90 (±7.279)	40	11.71	12.09
Year 4	2020	4.5	10.98 (±5.551)	34	10.67	11.29

*n*: Sample size; SD: Standard deviation; CI: Confidence interval (95%). Mean (SD) represents the average GAD-7 score with its standard deviation. The 95% CI is the range within which the true population mean is expected to fall with 95% confidence.

**Table 5:** ANOVA and Welch’s ANOVA Results for PHQ-9 Scores Across Time Periods (September 2020–September 2024; *n* = 44,676).

Source	Sum of Squares	df	Mean Square	F	Sig.	η^2^
Between Groups	130,571.759	3	43,523.920	946.144	<0.001	0.060
Within Groups	2,054,973.644	44,672	46.001	-	-	-
Total	2,185,545.403	44,675	-	-	-	-
	Welch’s F		df _1_	df _2_		Sig.
Welch	977.344		3	7898.668		<0.001

Note. df: Degrees of Freedom; F: F-ratio; Sig.: *p*-value indicating statistical significance; η^2^: Eta squared, a measure of effect size. df _1_ represents the degrees of freedom for the numerator (number of groups minus one), while df _2_ is adjusted based on the variance and sample sizes in each group.

**Table 6:** Post Hoc Comparisons of PHQ-9 Scores Across Time Periods Using Games–Howell Test.

Comparations	Mean Difference	SE	Sig.	95% CI Lower	95% CI Upper
Year 1–Year 2	−3.506	0.070	<0.001	−3.68	−3.33
Year 1–Year 3	−3.529	0.107	<0.001	−3.80	−3.25
Year 1–Year 4	−2.616	0.166	<0.001	−3.04	−2.19
Year 2–Year 3	−0.023	0.110	0.997	−0.31	0.26
Year 2–Year 4	0.890	0.167	<0.001	0.46	1.32
Year 3–Year 4	0.913	0.186	<0.001	0.43	1.39

Note. SE: Standard error of the mean difference; Sig.: *p*-value indicating statistical significance; CI: Confidence interval (95%). The 95% CI is the range within which the true population mean is expected to fall with 95% confidence.

**Table 7:** Pearson Correlation Between Anxiety (GAD-7) and Depression (PHQ-9) Across Time Periods.

Time Period	Number of Responses (*n*)		Pearson *r*	Sig.
	GAD-7	PHQ-9		
Total (Year 1–Year 4)	43,563	44,676	0.739	<0.001
Year 1	18,224	18,873	0.736	<0.001
Year 2	17,850	18,135	0.716	<0.001
Year 3	5515	5648	0.728	<0.001
Year 4	1974	2020	0.718	<0.001

Note. Pearson *r*: Pearson correlation coefficients; Sig.: *p*-value indicating statistical significance.

## Data Availability

The data that support the findings of this study are available on request from the corresponding author. The data are not publicly available due to privacy or ethical restrictions.

## Supplementary Materials

The following supporting information can be downloaded at: https://www.mdpi.com/article/10.3390/epidemiologia7020056/s1, Table S1. Mental health and well-being strategies of the ‘P5 Mental Health’ platform; Table S2. The number of responses submitted to the ‘P5 Mental Health’ platform between September 2020 and September 2024.

## References

[1] Nogueira-Leite D., Marques-Cruz M., Cruz-Correia R. (2024). Individuals’ attitudes toward digital mental health apps and implications for adoption in Portugal: Web-based survey. BMC Med. Inform. Decis. Mak..

[2] World Health Organization (2022). World Mental Health Report: Transforming Mental Health for All, W.H.O. https://www.who.int/publications/i/item/9789240049338.

[3] Global Burden of Disease Collaborative Network Global Burden of Disease Study 2019 (GBD 2019) Reference Life Table. http://ghdx.healthdata.org/record/ihme-data/global-burden-disease-study-2019-gbd-2019-reference-life-table.

[4] Hwang W.J., Ha J.S., Kim M.J. (2021). Research Trends on Mobile Mental Health Application for General Population: A Scoping Review. Int. J. Environ. Res. Public Health.

[5] Eisenstadt M., Liverpool S., Infanti E., Ciuvat R.M., Carlsson C. (2021). Mobile Apps That Promote Emotion Regulation, Positive Mental Health, and Well-being in the General Population: Systematic Review and Meta-analysis. JMIR Ment. Health.

[6] Gigantesco A., Fagnani C., Toccaceli V., Stazi M.A., Lucidi F., Violani C., Picardi A. (2019). The Relationship Between Satisfaction With Life and Depression Symptoms by Gender. Front. Psychiatry.

[7] Fergusson D.M., McLeod G.F.H., Horwood L.J., Swain N.R., Chapple S., Poulton R. (2015). Life satisfaction and mental health problems (18 to 35 years). Psychol. Med..

[8] Connell J., O’CAthain A., Brazier J. (2014). Measuring quality of life in mental health: Are we asking the right questions?. Soc. Sci. Med..

[9] Ohrnberger J., Fichera E., Sutton M. (2017). The relationship between physical and mental health: A mediation analysis. Soc. Sci. Med..

[10] Lombardo P., Jones W., Wang L., Shen X., Goldner E.M. (2018). The fundamental association between mental health and life satisfaction: Results from successive waves of a Canadian national survey. BMC Public Health.

[11] (2016). Gartner Inc. http://www.gartner.com.

[12] Salehan M., Negahban A. (2013). Social networking on smartphones: When mobile phones become addictive. Comput. Hum. Behav..

[13] Anthes E. (2016). Mental health: There’s an app for that. Nature.

[14] Watson A., Heyman I., Thomas S. (2020). Law enforcement and public mental health. J. Psychiatr. Ment. Health Nurs..

[15] Tinoco-González D., Fullana M.A., Torrents-Rodas D., Bonillo A., Vervliet B., Pailhez G., Farré M., Andión O., Perez V., Torrubia R. (2014). Conditioned Subjective Responses to Socially Relevant Stimuli in Social Anxiety Disorder and Subclinical Social Anxiety. Clin. Psychol. Psychother..

[16] Gokce H.H.Y., Dasdemir S., Kucukali C.I., Iplik E.S., Cakmakoglu B. (2020). G protein gene variants in schizophrenia. Arch. Clin. Psychiatry.

[17] Vigo D., Thornicroft G., Atun R. (2016). Estimating the true global burden of mental illness. Lancet Psychiatry.

[18] Firth J., Torous J., Nicholas J., Carney R., Pratap A., Rosenbaum S., Sarris J. (2017). The efficacy of smartphone-based mental health interventions for depressive symptoms: A meta-analysis of randomized controlled trials. World Psychiatry.

[19] Torous J., Myrick K.J., Rauseo-Ricupero N., Firth J. (2020). Digital Mental Health and COVID-19: Using Technology Today to Accelerate the Curve on Access and Quality Tomorrow. JMIR Ment. Health.

[20] Kalin N.H. (2020). The Critical Relationship Between Anxiety and Depression. Am. J. Psychiatry.

[21] Kessler R.C., Berglund P., Demler O., Jin R., Merikangas K.R., Walters E.E. (2005). Lifetime Prevalence and Age-of-Onset Distributions of DSM-IV Disorders in the National Comorbidity Survey Replication. Arch. Gen. Psychiatry.

[22] Levin M.E., Haeger J., Cruz R.A. (2018). Tailoring Acceptance and Commitment Therapy Skill Coaching in the Moment Through Smartphones: Results from a Randomized Controlled Trial. Mindfulness.

[23] Wind T.R., Rijkeboer M., Andersson G., Riper H. (2020). The COVID-19 pandemic: The ‘black swan’ for mental health care and a turning point for e-health. Internet Interv..

[24] Hollis C., Falconer C.J., Martin J.L., Whittington C., Stockton S., Glazebrook C., Davies E.B. (2016). Annual Research Review: Digital health interventions for children and young people with mental health problems—A systematic and meta-review. J. Child Psychol. Psychiatry.

[25] Naslund J.A., Aschbrenner K.A., Marsch L.A., Bartels S.J. (2016). The future of mental health care: Peer-to-peer support and social media. Epidemiol. Psychiatr. Sci..

[26] Firth J., Torous J., Nicholas J., Carney R., Rosenbaum S., Sarris J. (2017). Can smartphone mental health interventions reduce symptoms of anxiety? A meta-analysis of randomized controlled trials. J. Affect. Disord..

[27] Bakker D., Kazantzis N., Rickwood D., Rickard N. (2016). Mental Health Smartphone Apps: Review and Evidence-Based Recommendations for Future Developments. JMIR Ment. Health.

[28] Di Carlo F., Sociali A., Picutti E., Pettorruso M., Vellante F., Verrastro V., Martinotti G., di Giannantonio M. (2020). Telepsychiatry and other cutting-edge technologies in COVID-19 pandemic: Bridging the distance in mental health assistance. Int. J. Clin. Pract..

[29] Czeisler M.É., Howard M.E., Rajaratnam S.M.W. (2021). Mental Health During the COVID-19 Pandemic: Challenges, Populations at Risk, Implications, and Opportunities. Am. J. Health Promot..

[30] Robinson E., Sutin A.R., Daly M., Jones A. (2021). A systematic review and meta-analysis of longitudinal cohort studies comparing mental health before versus during the COVID-19 pandemic in 2020. J. Affect. Disord..

[31] Holmes E.A., O’Connor R.C., Perry V.H., Tracey I., Wessely S., Arseneault L., Ballard C., Christensen H., Silver R.C., Everall I. (2020). Multidisciplinary research priorities for the COVID-19 pandemic: A call for action for mental health science. Lancet Psychiatry.

[32] Xiong J., Lipsitz O., Nasri F., Lui L.M.W., Gill H., Phan L., Chen-Li D., Iacobucci M., Ho R., Majeed A. (2020). Impact of COVID-19 pandemic on mental health in the general population: A systematic review. J. Affect. Disord..

[33] Vindegaard N., Benros M.E. (2020). COVID-19 pandemic and mental health consequences: Systematic review of the current evidence. Brain Behav. Immun..

[34] Prati G., Mancini A.D. (2021). The psychological impact of COVID-19 pandemic lockdowns: A review and meta-analysis of longitudinal studies and natural experiments. Psychol. Med..

[35] Twenge J.M., Joiner T.E. (2020). Mental distress among U.S. adults during the COVID-19 pandemic. J. Clin. Psychol..

[36] Vahia I.V., Jeste D.V., Reynolds C.F. (2020). Older Adults and the Mental Health Effects of COVID-19. JAMA.

[37] Silva Moreira P., Ferreira S., Couto B., Machado-Sousa M., Fernández M., Raposo-Lima C., Sousa N., Picó-Pérez M., Morgado P. (2021). Protective Elements of Mental Health Status during the COVID-19 Outbreak in the Portuguese Population. Int. J. Environ. Res. Public Health.

[38] Monaco F., Vignapiano A., Piacente M., Pagano C., Mancuso C., Steardo L., Marenna A., Farina F., Petrillo G., Leo S. (2024). An advanced Artificial Intelligence platform for a personalised treatment of Eating Disorders. Front. Psychiatry.

