# Supplementary material for: P5 Mental Health Platform: A Digital Solution to Monitor Anxiety and Depression Symptoms in the General Portuguese Population
Source: Epidemiologia (Basel). 2026 Apr 20;7(2):56. doi: 10.3390/epidemiologia7020056 (PMC13114630; doi:10.3390/epidemiologia7020056)
Supplement: Supplementary file 1 [file epidemiologia-07-00056-s001.zip › epidemiologia-3513616-supplementary.pdf]

Supplementary Material

Table S1. Mental health and well-being strategies of the ‘P5 Mental Health’ platform.

<table>
<tr><th colspan="2">Wellness activities</th></tr>
<tr><td colspan="2">Watch a movie or series; Watch/follow some sport; Cook; Watch fun and funny videos; Dance; Solve sudoku, puzzles, or similar games; Chat with someone close; Listen to music; See old photos; Learn a foreign language; Develop a new hobby; Read a book; Meditate; Draw or paint; Take a walk; Practice physical exercise; Sunbathing; Make a short list of things you are grateful for; Do some gardening.</td></tr>
<tr><td colspan="2">Emotional Regulation</td></tr>
<tr><td>Focus on Reality</td><td>Identify 5 things you can see around you; Identify 3 things you can hear; Name 5 different cereal brands; Name 5 different countries; Identify 3 things you can smell; Name 5 different animals that fly.</td></tr>
<tr><td>Functional Thoughts</td><td>"I cannot control everything"; “I can handle it”; “A thought is just a thought and only has the importance I attribute to it”; “Just thinking that something bad is going to happen doesn’t mean that it will happen”; "What I'm feeling may be unpleasant, but it won't last forever"; “Challenges and difficulties bring me opportunities for learning and growth”; “I am safe”; “A step back does not mean going back to square one”.</td></tr>
<tr><td>Change of Perspective</td><td>Are your thoughts based on facts or interpretations?; Are there other ways of interpreting this situation?; What is the evidence that supports your thinking?; Is this situation as serious as you're thinking?; How would someone more optimistic interpret this situation?; What is the worst outcome you can expect and how likely is it to happen?; What can you do to resolve the issue?; Is there anything useful you can learn from this situation that will allow you to better handle similar situations in the future?.</td></tr>
<tr><td colspan="2">Thinking Patterns</td></tr>
<tr><td colspan="2">“What if?”: Constant questions and doubts like “What if…?”.
Negative filter: Focus almost exclusively on what is negative.
Focus on judgment: Everything is considered in terms of evaluations: “good/bad”, “superior/inferior”.
Generalization: Perceiving a global pattern of negative things based on incidents only.
Mind reading: Assuming you know what others think without having proof of it.
Dichotomous thinking: All-or-nothing thinking.
Personalization: Disproportionately blaming oneself for negative events.
Emotional reasoning: Feelings guide the interpretation of reality.
Label: Attributing negative characteristics to self and others.
Catastrophizing: Predicting that something unacceptable and unbearable will happen.</td></tr>
<tr><td colspan="2">Relaxation</td></tr>
<tr><td colspan="2">Progressive Muscle Relaxation (audio: 5.28 sec.)
Diaphragmatic Breathing (audio: 2.09 sec.)
Mindfulness – Attention to Breathing (audio: 6.58 sec.)
Mindfulness – Body Scan (audio: 13.15 sec.)
Mindfulness – Exploring Difficult Feelings and Thoughts (audio: 6.30 sec.)</td></tr>
<tr><td colspan="2">Sleep</td></tr>
<tr><td>Activities to ‘Switch Off’</td><td>Allowing yourself to have a quiet moment before bedtime. This can help your mind and body relax and serves as a transition between the energetic activities of the day and bedtime.
Here are some suggested activities: Choose clothes for the next day; Watch a movie, series, or other TV program that helps you relax; Prepare meals for the next day; Do some stretches to relax your muscles; Listen to calm and relaxing music; Write a diary; Take the dog for a walk; Organize some papers, photos, books, or others; To have a bath; Take care of pets (feed them, brush them, etc.).</td></tr>
<tr><td>Activities when you cannot sleep</td><td>Getting out of bed when you can't sleep to do some relaxing activity helps your body associate the bed with the specific function of sleep.
Here are some suggestions for small activities and chores to do when you can't sleep: Organize the e-mail (e.g., delete spam, organize folders); Reading a magazine or other light reading material; Create a list of activities you would like to do on weekends or vacations; Pack and organize clothes; Make a shopping list for the week; Play some game that distracts, but is not competitive; Choose a small drawer to store or organize; Plan the meal menu for the week.</td></tr>
<tr><td>Sleep hygiene recommendations</td><td>1) If, after going to bed, you have not fallen asleep after 20 minutes, get up and try to do some light activity.</td></tr>
</table>

	2) Avoid looking at the clock - constantly noticing how long you have been awake will increase worry and anxiety about bedtime.
	3) Disconnect from technologies (e.g., television, computer, cell phone, etc.) at least 30 minutes before going to bed. Brightness from screens is not beneficial for sleep.
	4) If you find yourself worrying too much about not being able to fall asleep, try to put those thoughts and worries into perspective.
	5) Try to maintain a comfortable environment at bedtime, considering aspects such as light, temperature, and noise.
	6) If you need to take a nap, do not extend it for more than 30 minutes and avoid taking it at the end of the day, as it may interfere with the quality and cycles of sleep.
	7) Practice physical exercise regularly. However, do not do this close to bedtime as it could have a negative impact on sleep.
	8) Maintain some regular routines, namely regarding waking up and bedtime. Establishing a relaxing bedtime routine is also essential.
	9) Avoid consuming alcohol or caffeine from the end of the day.

Table S2. The number of responses submitted to the 'P5 Mental Health' platform between September 2020 and September 2024.

Year	Months	Number of Responses		
		GAD-7	PHQ-9	Total
**2020 (n)**	Set	4046	4099	4100
	Oct	694	708	708
	Nov	190	195	195
	Dec	101	103	103
Total of the year (n)		5031	5105	5106
2021 (n)	Jan	154	161	161
	Feb	10022	10349	10556
	Mar	1799	1896	1964
	Apr	392	457	476
	May	405	472	494
	Jun	175	181	189
	Jul	120	124	126
	Aug	126	128	134
	Set	177	203	219
	Oct	258	264	272
	Nov	225	224	237
	Dec	146	145	152
Total of the year (n)		13999	14604	14980
2022 (n)	Jan	215	220	229
	Feb	944	980	1025
	Mar	6600	6701	7179
	Apr	1733	1763	1827
	May	530	546	572
	Jun	5106	5142	5360
	Jul	1464	1494	1549
	Aug	452	453	464
	Set	853	866	885
	Oct	1221	1258	1297
	Nov	431	438	451
	Dec	327	336	337
Total of the year (n)		19876	20197	21175
2023 (n)	Jan	610	625	625
	Feb	401	409	409
	Mar	370	380	380
	Apr	333	339	339
	May	316	331	332
	Jun	285	291	291
	Jul	195	198	198
	Aug	173	177	177
	Set	188	189	189
	Oct	419	429	429
	Nov	193	194	194
	Dec	163	166	166
Total of the year (n)		3646	3728	3729
2024 (n)	Jan	237	242	242
	Feb	111	112	112
	Mar	127	130	130
	Apr	170	177	177
	May	133	141	141
	Jun	78	80	80
	Jul	49	49	49
	Aug	48	50	50
	Set	58	61	61
Total of the year (n)		1011	1042	1042

Total between 2020-2024 (n)	43563	44676	46032
